# Editorial: Employment sustainability and teaching/learning techniques in higher education institutions

**DOI:** 10.3389/fpsyg.2024.1373916

**Published:** 2024-03-07

**Authors:** Arsalan Mujahid Ghouri, Muhammad Awais Bhatti, Ariff Syah Juhari

**Affiliations:** ^1^School of Business, London South Bank University, London, United Kingdom; ^2^Department of Management, King Faisal University, Al-Ahsa, Saudi Arabia; ^3^College of Business Administration, Prince Sultan University, Riyadh, Saudi Arabia

**Keywords:** employment sustainability, higher education institutions (HEIs), teaching techniques, learning techniques in soft robotics, Malaysia, China

## Aims and objectives of the Research Topic

The intersection of education and employability takes center stage in our ever-evolving global landscape. This Research Topic delves into the intricate dance between higher education institutions (HEIs) and industry demands, aiming to unearth sustainable solutions to bridge the skills gap and prepare graduates for the complexities of the modern workforce.

The initial call for this Research Topic addressed the ongoing challenges faced by employers who seek competent adaptable employees. It pinpointed the perceived shortcomings of the education system, urging HEIs to align their curricula with the dynamic needs of industries. The emphasis on intercultural and interdisciplinary approaches was underscored, recognizing the importance of preparing graduates for a multicultural rapidly changing job market.

## Outcome

This Research Topic provides opportunities for educationalists and researchers to shed light on the challenges and opportunities in the education sector. The first article (Park), exploring the re-entry experiences of kinesiology professionals, brings to light the challenges and triumphs encountered upon returning to their home country. The findings resonate with the re-acculturation theory, emphasizing the importance of addressing issues faced by professionals re-entering academia. The second article (Jamaludin et al.) focuses on reshaping the curriculum for applied industry-focused training (AiF). It highlights the current vagueness surrounding AiF and proposes a framework rooted in an industry-based curriculum, employability skills, and international best practices. The framework aims to guide ministries, educators, and industries in enhancing AiF practices for a more responsive and effective outcome.

The third article (Wang et al.) delves into the demographic migration of young teachers from Taiwan to China. Employing a push-pull-mooring model, it unravels the factors influencing the decision of young teachers, shedding light on the dynamic interplay of forces contributing to this cross-border academic migration. The fourth article (Zhou et al.) explores the intricate relationships between emotional intelligence, career decision-making self-efficacy, and employability. It highlights the significance of emotional intelligence in enhancing employability, emphasizing how career decision-making self-efficacy plays a pivotal role in shaping both.

## Conclusion

As we weave through these diverse articles, a cohesive narrative emerges—an urgent need for HEIs to adapt and innovate in tandem with industry demands. The call for sustainable education practices echoes throughout, urging institutions to embrace multicultural perspectives, interdisciplinary approaches, and a keen focus on developing not only technical skills but also the emotional intelligence and self-efficacy crucial for navigating the modern workforce.

## Moving forward

This Research Topic serves as a compass, guiding HEIs, policymakers, and industry stakeholders toward collaborative strategies that nurture a skilled, adaptable, and culturally aware workforce. The synthesis of these articles invites us to reflect on the symbiotic relationship between education and employability, urging us to bridge the gap and foster a future in which graduates seamlessly transition into the workforce, equipped to thrive in our multicultural and ever-changing world.

## Author contributions

AG: Conceptualization, Writing—original draft, Writing—review & editing. MB: Conceptualization, Validation, Writing—original draft, Writing—review & editing. AJ: Validation, Writing—original draft, Writing—review & editing.

